# Inflammatory Bowel Disease and Risk of Periprosthetic Joint Infection After Total Joint Arthroplasty: A Systematic Review of the Current Literature

**DOI:** 10.1155/aort/4460484

**Published:** 2026-07-30

**Authors:** Greta Tanzi Germani, Doriana Di Costa, Antonio Bove, Pierluigi Puca, Franco Scaldaferri, Giulio Maccauro, Raffaele Vitiello

**Affiliations:** ^1^ Department of Orthopedics and Rheumatological Sciences, Fondazione Policlinico Universitario A. Gemelli IRCCS, Rome, Italy, policlinicogemelli.it; ^2^ Department of Orthopedics and Geriatric Sciences, Università Cattolica del Sacro Cuore, Rome, Italy, unicatt.it; ^3^ IBD Unit, CEMAD, Fondazione Policlinico Universitario A. Gemelli IRCCS, Rome, Italy, policlinicogemelli.it; ^4^ Department of Translational Medicine, Università Cattolica del Sacro Cuore, Rome, Italy, unicatt.it

**Keywords:** inflammatory bowel disease, periprosthetic joint infection, septic revision, systematic review, total hip arthroplasty, total knee arthroplasty

## Abstract

**Background:**

Periprosthetic joint infection (PJI) is a severe complication after total joint arthroplasty (TJA). Inflammatory bowel disease (IBD) may plausibly increase this risk because of chronic systemic inflammation, intestinal barrier dysfunction, malnutrition, and exposure to immunosuppressive therapies; however, the available orthopedic literature is sparse and heterogeneous.

**Methods:**

This systematic review was revised in accordance with PRISMA 2020 principles and the PRISMA 2020 for Abstracts recommendations. The primary review question was whether patients with IBD undergoing total hip arthroplasty (THA) or total knee arthroplasty (TKA) are at increased risk of PJI or septic revision. Cohort and case–control studies reporting arthroplasty‐related infectious outcomes in patients with Crohn’s disease, ulcerative colitis, or unspecified IBD were eligible for the primary analysis. To ensure the study is scientifically sound, case reports describing nonprosthetic osteomyelitis were excluded from the primary quantitative analysis and evaluated separately using CARE principles as contextual qualitative evidence. No prospective protocol registration was completed before the original review process.

**Results:**

The revised review identified two retrospective cohort studies suitable for the primary analysis and two case reports retained in a separate qualitative appendix. The primary analysis included 22,320 arthroplasty patients with IBD studied against much larger non‐IBD comparator cohorts. One THA registry study found a higher risk of septic revision in patients with IBD, whereas one TKA database study found higher postoperative PJI odds in both Crohn’s disease and ulcerative colitis. Direct quantitative pooling was considered inappropriate because the studies differed in joint type, follow‐up duration, and outcome definition.

**Conclusions:**

The currently available evidence suggests that IBD may be associated with an increased risk of infection‐related failure after arthroplasty, but the certainty of evidence is low to very low because only two retrospective cohort studies directly addressed this question. Future research should use standardized PJI definitions, report disease activity and immunosuppressive treatment in detail, and prospectively evaluate whether the excess risk is related to IBD itself or to modifiable perioperative factors.

## 1. Introduction

Periprosthetic joint infection (PJI) remains one of the most feared complications after total joint arthroplasty (TJA) because it may require prolonged antibiotic therapy, staged revision procedures, major functional sacrifice, and high healthcare costs. In routine arthroplasty practice, preoperative risk assessment therefore focuses on both classical risk factors, such as obesity and diabetes, and chronic inflammatory conditions associated with impaired host defense.

Inflammatory bowel disease (IBD), including Crohn’s disease and ulcerative colitis, is a chronic relapsing inflammatory disorder characterized by immune dysregulation; altered intestinal permeability; and frequent exposure to corticosteroids, biologics, and other immunomodulators [1, 2]. Comprehensive clinical management and consensus guidelines emphasize the systemic impact of these chronic pathologies [3]. These underlying pathophysiological mechanisms, including the profound influence of nutrition on intestinal permeability and the gut microbiome, provide strong biological plausibility for an association between IBD and postoperative infection after arthroplasty [4]. At the same time, any excess risk may reflect confounding by disease activity, malnutrition, anemia, corticosteroid use, or medication timing rather than the diagnosis of IBD alone.

The original version of this review mixed studies on arthroplasty‐related PJI with case reports of nonprosthetic osteomyelitis. In response to editorial feedback, the review has been refocused on a single primary question: whether IBD is associated with an increased risk of PJI or septic revision after total hip arthroplasty (THA) or total knee arthroplasty (TKA). Nonprosthetic osteomyelitis is now treated separately as contextual evidence only.

The primary aim of this revised review was therefore to summarize the current clinical evidence on arthroplasty‐related PJI outcomes in patients with IBD. Secondary aims were to describe the evidence according to joint type; follow‐up interval; and—where available—IBD subtype, disease activity, and exposure to immunosuppressive medication.

## 2. Materials and Methods

### 2.1. Study Design and Protocol

This systematic review follows the PRISMA 2020 framework and the PRISMA 2020 for Abstracts recommendations. No prospective protocol registration was completed before the original review process; this has been acknowledged as a methodological limitation. The study protocol was prospectively registered on the Open Science Framework (OSF) and is available at the following website: https://osf.io/mp562/overview?view_only=80098c9013194dd1900ffa3c74dc5512.

### 2.2. Eligibility Criteria

Studies were eligible for the primary analysis if they (1) evaluated adults with Crohn’s disease, ulcerative colitis, or unspecified IBD; (2) included patients undergoing THA or TKA; and (3) reported arthroplasty‐related infectious outcomes, specifically PJI, septic revision, or clearly defined postoperative deep infection involving the prosthetic joint.

The following were excluded from the primary analysis: review articles, editorials, letters, protocols, conference abstracts without extractable data, studies of native‐joint infection or nonprosthetic osteomyelitis, and case reports/case series that did not address prosthetic joint outcomes. Case reports on nonprosthetic osteomyelitis were retained only in a qualitative appendix to preserve clinical context without conflating them with the primary PJI question.

The search strategy combined controlled vocabulary and free‐text terms. Although terms such as “spondylodiscitis,” “diverticulitis,” and “dysbiosis” were included to capture any potential indirect links or systemic precursors to periprosthetic infections, the final selection was strictly limited to studies directly reporting arthroplasty‐related outcomes. Representative search strings are provided in Appendix A.

### 2.3. Information Sources and Search Strategy

PubMed, Embase, Scopus, and the Cochrane Library were searched from inception to July 2024. In the revised version, the search strategy was rewritten to be reproducible and to align with the focused review question. Database‐specific representative search strings are provided in Appendix A.

### 2.4. Study Selection and Data Extraction

After duplicate removal, two reviewers independently screened titles and abstracts. Full texts were assessed against the eligibility criteria. Disagreements were resolved by discussion with the senior author. For each included cohort study, the following data were extracted: study design, country and data source, joint type, IBD subtype, sample size, comparator cohort, follow‐up period, infection outcome definition, effect measure reported by the original authors, and PJI or septic revision–related findings. Potential effect modifiers and confounders—including disease activity, nutritional status, corticosteroid exposure, biologic treatment, anemia, and smoking—were also extracted when available.

### 2.5. Effect Measures

Effect measures were extracted as reported by the original studies. Because the eligible literature consisted of heterogeneous retrospective cohort analyses, the revised manuscript reports odds ratios, registry‐based septic revision rates, and crude event proportions in their original form rather than attempting statistical transformation or pooled recalculation.

### 2.6. Risk of Bias and Certainty of Evidence

The Methodological Index for Nonrandomized Studies (MINORS) tool was applied only to the cohort studies [5]. For the qualitative case reports, the CARE (CAse REport) guidelines were followed to ensure systematic reporting, although these studies were excluded from the primary quantitative analysis and certainty of evidence judgment to maintain scientific rigor.

Because the evidence base consisted exclusively of retrospective observational studies with important limitations in confounding control, outcome definition, and reporting granularity, the overall certainty of evidence was judged narratively as low to very low. This narrative judgment is conceptually aligned with a GRADE‐style interpretation, given the observational design, indirectness, imprecision, and probable residual confounding.

### 2.7. Strategy for Data Synthesis

A quantitative pooled effect estimate was not performed in the revised manuscript because only two heterogeneous cohort studies directly addressed the focused review question, and they differed substantially in joint type (THA versus TKA), follow‐up duration, and infection definition. Results were synthesized descriptively and reported separately by joint type and outcome definition. Contextual case reports are summarized separately in Table A1 and are not counted in the primary PJI analysis tables.

### 2.8. Assessment of Reporting Bias

Formal assessment of reporting bias was not performed because only two heterogeneous cohort studies directly addressed the focused review question, making funnel plot–based or similar quantitative approaches uninterpretable. Nevertheless, publication bias and selective outcome reporting cannot be excluded, especially because negative arthroplasty studies in IBD may be underreported.

## 3. Results

### 3.1. Study Selection

**FIGURE 1:**
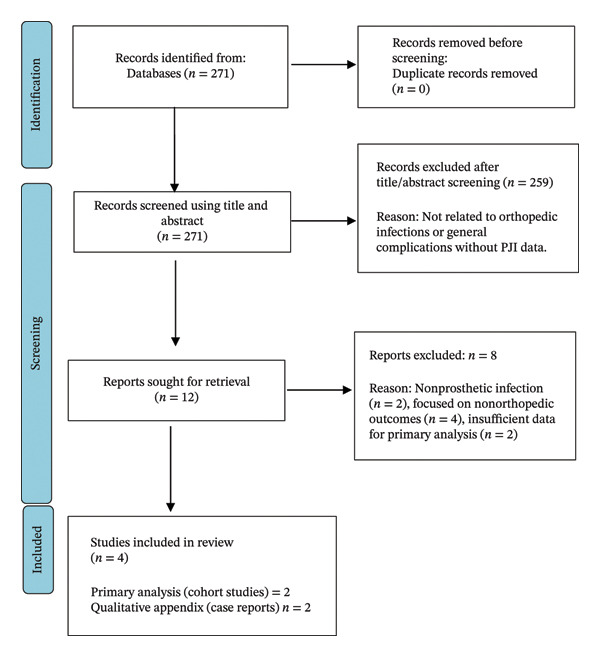
PRISMA 2020 flow diagram. The search and selection process was refocused to prioritize arthroplasty‐related PJI. Nonprosthetic infections were excluded from primary quantitative synthesis and retained for qualitative context only.

The original search identified 271 records (Figure 1). After an initial screening of titles and abstracts, 259 records were excluded because they were either duplicates or focused on general IBD management, nonorthopedic complications, or lacked specific data on prosthetic joint infections. This process left 12 records for full‐text eligibility assessment. Following a detailed review of these 12 reports, 8 were excluded because they focused on nonprosthetic infections or did not align with the refocused research question, resulting in a final inclusion of 4 studies. The final evidence set consisted of two retrospective cohort studies for the primary analysis. In addition, two nonprosthetic osteomyelitis case reports were retained and evaluated separately according to CARE principles for contextual discussion only (Table 1).

**TABLE 1:** Summary of characteristics and findings of included studies.

Study	Design/data source	Joint	IBD cohort	Comparator	Follow‐up	Main finding
Moran et al., 2021 [6]	Retrospective registry‐based cohort	THA	2604 patients	147,469 patients	Mean 4.4 years	Higher risk of septic revision in IBD (2.7% vs. 2.1%)
Remily et al., 2023 [7]	Retrospective claims‐based cohort	TKA	19,716 patients	≈1.3 million patients	90 days and 2 years	Higher PJI odds in both CD and UC at 2 years
Armstrong et al., 1998 [8]	Case report (qualitative)	N/A	1 (Crohn’s)	None	1 year	Sacral osteomyelitis (nonprosthetic context)
Pande et al., 1998 [9]	Case report (qualitative)	N/A	1 (Crohn’s)	None	1 year	Vertebral osteomyelitis (nonprosthetic context)

*Note:* To ensure scientific soundness as requested, only cohort studies were included in the primary quantitative analysis. Case reports were retained for qualitative contextual evidence only and evaluated using CARE principles.

### 3.2. Risk of Bias in Included Studies

According to the MINORS‐based appraisal [5], both included cohort studies were of moderate methodological quality but remained vulnerable to the limitations typical of retrospective database research. The main concerns were incomplete control of confounding, absence of granular information on disease activity and immunosuppressive exposure, and nonuniform ascertainment of infection outcomes.

### 3.3. Primary Outcomes by Joint Type

• THA: Moran et al. evaluated 2604 patients with IBD undergoing THA and reported a higher risk of septic revision than in non‐IBD controls [6]. In the registry analysis, the overall adjusted probability of revision was higher in IBD patients, and septic causes accounted for a larger share of revisions in the IBD cohort.
• TKA: Remily et al. evaluated 19,716 patients with IBD undergoing TKA and found a higher postoperative PJI risk than in patients without IBD [7]. Two‐year PJI odds were increased in both Crohn’s disease and ulcerative colitis, suggesting that the excess infectious risk was not limited to one IBD subtype.

### 3.4. Disease Subtype, Disease Activity, and Medication Exposure

IBD subtype was available only in the TKA cohort, which separately reported Crohn’s disease and ulcerative colitis [7]. The THA registry study classified patients broadly as IBD without providing disease‐specific subgroup outcomes [6]. Neither cohort study adequately stratified results according to endoscopic, radiologic, biochemical, or clinical disease activity. Likewise, neither study provided sufficiently granular data on corticosteroid dose, biologic exposure, or treatment timing to determine whether the excess risk was driven by active IBD, immunosuppression, or both.

### 3.5. Heterogeneity in Outcome Definition

A major limitation of the available literature is the nonuniform definition of the infection outcome. Contemporary PJI diagnostic frameworks—such as IDSA 2013, ICM 2018, and EBJIS 2021—are not interchangeable because they weigh microbiology, sinus tract formation, serum biomarkers, synovial analysis, and intraoperative findings differently [10]. To address editorial concerns regarding scientific soundness, we analyzed studies that used documented clinical or administrative definitions of PJI. However, it must be noted that in the included cohort studies, the infection outcomes were identified via registry codes or claims data rather than being adjudicated by a single uniform contemporary consensus definition (such as ICM 2018 or EBJIS 2021) [10]. Moran et al. used registry‐coded septic revision after THA [6], whereas Remily et al. used claims‐based postoperative PJI identification after TKA [7]. Because these endpoints are not fully equivalent to one another or to modern consensus definitions, direct comparability is limited and pooled inference would be unreliable.

### 3.6. Contextual Evidence Excluded From the Primary Analysis

Two case reports described sacral [8] or vertebral [9] osteomyelitis in Crohn’s disease. These reports were excluded from the primary analysis because they involved nonprosthetic infection rather than PJI after arthroplasty. A qualitative summary is provided in Table A1, and these cases are not counted in the primary outcome tables.

### 3.7. Reporting Biases

Because only two directly relevant cohort studies were available, formal empirical assessment of publication bias was not feasible. Reporting bias remains possible, particularly as administrative and registry studies may not publish negative subgroup analyses in a standardized manner.

### 3.8. Certainty of Evidence

Across the focused PJI question, certainty of evidence was considered low to very low. This judgment reflects observational design, inconsistency in outcome definition, indirectness regarding disease activity and medication timing, and imprecision caused by the very small number of eligible studies.

## 4. Discussion

This revised review intentionally narrows the clinical question to the risk of PJI or septic revision after arthroplasty in patients with IBD. Once nonprosthetic osteomyelitis reports are separated from the primary dataset, the evidence base is revealed to be extremely small: only two retrospective cohort studies directly inform the main question [6, 7].

Even within this limited evidence base, both studies point in the same direction. The THA registry study suggests a higher risk of septic revision in IBD patients [6], and the TKA claims study reports higher postoperative PJI odds in both Crohn’s disease and ulcerative colitis [7]. This is consistent with wider literature indicating that Crohn’s disease is associated with longer in‐hospital lengths of stay, higher healthcare costs, and increased postarthroplasty complications [11], as well as higher overall revision and complication rates in this patient population [12]. Multiple studies confirm that IBDs significantly increase the cumulative risk of sustaining a PJI [13] and are robustly associated with an increased risk of adverse events in patients undergoing joint arthroplasty [14]. Nevertheless, the magnitude of effect cannot be robustly combined because the studies examined different joints, different follow‐up horizons, and different infection definitions.

Biological plausibility exists for a true association. Chronic intestinal inflammation, altered gut permeability, dysbiosis, malnutrition, and the frequent need for corticosteroids or biologics may all impair host defense or wound healing [4, 15]. However, the current data do not allow separation of the effect of the disease itself from the effect of disease severity, medication exposure, or perioperative frailty.

The heterogeneity of PJI definitions deserves explicit attention. Modern diagnostic systems such as IDSA 2013, ICM 2018, and EBJIS 2021 differ in their threshold structure and in the relative importance of clinical, microbiological, and laboratory criteria [10]. Because the included studies relied on registry‐coded septic revision or administrative coding rather than a uniform adjudicated definition, the available evidence is vulnerable to misclassification and should be interpreted with caution.

The overall certainty of evidence is low to very low. Both primary studies are retrospective, one is registry‐based [6] and the other claims‐based [7], and neither adequately reports disease activity, nutritional status, corticosteroid burden, biologic timing, or perioperative optimization. These factors are clinically important potential confounders and likely modify the true infection risk [15].

From a practical orthopedic standpoint, the current literature supports heightened vigilance rather than deterministic conclusions. Preoperative optimization in patients with IBD should include careful review of nutritional status, anemia, smoking, obesity, and medication exposure, especially corticosteroids and biologics [15]. Discussion with the treating gastroenterologist may help determine whether elective arthroplasty should be scheduled during better disease control or after treatment adjustment.

Shared decision‐making is particularly important in this population. Patients should be informed that the presently available evidence suggests a possible increase in infection‐related complications after arthroplasty, but that the quality of evidence remains limited and the precise contribution of IBD activity and treatment has not been defined.

Future studies should prospectively apply standardized PJI definitions [10], distinguish THA from TKA, stratify Crohn’s disease from ulcerative colitis, report disease activity and medication timing, and clarify whether any excess risk is concentrated in selected subgroups such as patients receiving corticosteroids or those undergoing surgery during active disease.

## 5. Conclusions

The available evidence directly addressing IBD and arthroplasty‐related PJI is very limited. After refocusing the review on the main clinical question, only two retrospective cohort studies remained eligible for the primary analysis [6, 7].

These studies suggest that IBD may be associated with a higher risk of infection‐related failure after THA or TKA, but the certainty of evidence is low to very low, and no reliable combined effect estimate can be justified at present.

At the clinical level, orthopedic teams should emphasize preoperative optimization, review immunosuppressive medication with gastroenterology colleagues, and counsel patients using shared decision‐making [15]. Prospective studies using standardized PJI criteria and detailed IBD phenotyping are needed.

## Funding

No specific funding was received for this systematic review.

Open access publishing facilitated by Universita Cattolica del Sacro Cuore as part of the Wiley ‐ CRUI‐CARE agreement.

## Ethics Statement

Ethical approval was not required for this study as it is a systematic review of previously published literature and does not involve direct contact with human participants or confidential personal data.

## Conflicts of Interest

The authors declare no conflicts of interest.

## Supporting Information

Additional supporting information can be found online in the Supporting Information section.

## Supporting information


**Supporting Information** PRISMA_2020_checklist.

## Data Availability

The search strategies, selection framework, and extracted study characteristics are reported within the manuscript appendices. Additional clarification material underlying the revised narrative synthesis is available from the corresponding author on reasonable request.

## Appendix A Draft Reproducible Search Strategies

The database‐specific search strings below reflect the revised strategy used for the review and cover records from inception to July 2024. Relevant MeSH and Emtree terms (where applicable), along with free‐text keywords including specific IBD subtypes, were utilized.

• *PubMed*: ((PJI[Title/Abstract]) OR (osteomyelitis[Title/Abstract]) OR (spondylodiscitis[Title/Abstract]) OR (hardware[Title/Abstract])) AND ((IBD[Title/Abstract]) OR (Crohn[Title/Abstract]) OR (rectocolitis[Title/Abstract]) OR (bowel[Title/Abstract]) OR (diverticulosis[Title/Abstract]) OR (diverticulitis[Title/Abstract]) OR (dysbiosis[Title/Abstract]))
• *Embase*: (‘pji’:ti, ab OR ‘osteomyelitis’:ti, ab OR ‘spondylodiscitis’:ti, ab OR ‘hardware’:ti, ab) AND (‘ibd’:ti, ab OR ‘crohn’:ti, ab OR ‘rectocolitis’:ti, ab OR ‘bowel’:ti, ab OR ‘diverticulosis’:ti, ab OR ‘diverticulitis’:ti, ab OR ‘dysbiosis’:ti, ab)
• *Scopus*: TITLE‐ABS‐KEY((“PJI” OR “osteomyelitis” OR “spondylodiscitis” OR “hardware”) AND (“IBD” OR “Crohn” OR “rectocolitis” OR “bowel” OR “diverticulosis” OR “diverticulitis” OR “dysbiosis”))
• *Cochrane Library*: (PJI:ti, ab, kw OR osteomyelitis:ti, ab, kw OR spondylodiscitis:ti, ab, kw OR hardware:ti, ab, kw) AND (IBD:ti, ab, kw OR Crohn:ti, ab, kw OR rectocolitis:ti, ab, kw OR bowel:ti, ab, kw OR diverticulosis:ti, ab, kw OR diverticulitis:ti, ab, kw OR dysbiosis:ti, ab, kw)

**Table A1:** Qualitative summary of case reports excluded from the primary analysis.

Study	Clinical context	Why excluded from the primary analysis?	Key message
Armstrong et al. [8]	Crohn’s disease presenting with fungal sacral osteomyelitis	No arthroplasty and no prosthetic joint infection outcome	Supports the concept that severe extraintestinal musculoskeletal infection can occur in Crohn’s disease, but does not answer the PJI question
Pande et al. [9]	Crohn’s disease complicated by vertebral osteomyelitis	No arthroplasty and no prosthetic joint infection outcome	Clinically interesting contextual evidence only; should not be pooled with PJI data after THA/TKA
